# Metabolic Improvements Following Upper Airway Surgery in Obstructive Sleep Apnea: Association of Airway Improvement with Insulin Resistance

**DOI:** 10.3390/jcm15124825

**Published:** 2026-06-21

**Authors:** Chia-Chen Lin, Wan-Ni Lin, Li-Jen Hsin, Ming-Shao Tsai, Li-Ang Lee, Hsueh-Yu Li

**Affiliations:** 1 Department of Otorhinolaryngology, Head and Neck Surgery, New Taipei Municipal Tucheng Hospital, New Taipei City 23652, Taiwan; lydia05025137@hotmail.com; 2 Department of Otorhinolaryngology, Head and Neck Surgery, Sleep Center, Linkou Chang Gung Memorial Hospital, Taoyuan City 333423, Taiwan; wannilin@hotmail.com (W.-N.L.); lijen.hsin@gmail.com (L.-J.H.); 5738@cgmh.org.tw (L.-A.L.); 3 School of Medicine, Chang Gung University, Taoyuan City 33302, Taiwan; b87401061@cgmh.org.tw; 4 Department of Otorhinolaryngology, Head and Neck Surgery, Chiayi Chang Gung Memorial Hospital, Chiayi 613, Taiwan; 5 School of Medicine, National Tsin Hua University, Hsinchu 300044, Taiwan

**Keywords:** obstructive sleep apnea, insulin resistance, HOMA-IR, diabetes, metabolic outcomes, airway obstruction

## Abstract

**Background:** Obstructive sleep apnea (OSA) is increasingly recognized as a systemic disorder associated with insulin resistance and elevated risk of type 2 diabetes. While continuous positive airway pressure (CPAP) is the standard therapy, its long-term metabolic benefits remain inconsistent. The metabolic impact of upper airway surgery is less well defined. **Methods:** In this retrospective study, 49 patients with polysomnography-confirmed OSA who underwent upper airway surgery were evaluated. Respiratory and metabolic parameters—including apnea–hypopnea index (AHI), fasting plasma glucose, fasting insulin, glycated hemoglobin (HbA1c), and homeostatic model assessment for insulin resistance (HOMA-IR)—were assessed preoperatively and at 6 months postoperatively. Associations between changes in AHI (ΔAHI) and insulin resistance (ΔHOMA-IR) were analyzed using correlation and receiver operating characteristic (ROC) analyses. **Results:** Significant improvements were observed in both respiratory and metabolic parameters. AHI decreased from 46.6 ± 25.8 to 20.7 ± 14.1 events/h (*p* < 0.001). Fasting plasma glucose, insulin levels, and HOMA-IR were significantly reduced postoperatively (all *p* < 0.05), while HbA1c showed a downward trend. Reduction in AHI was moderately correlated with improvement in insulin resistance (r = 0.527, *p* < 0.001). ROC analysis demonstrated modest discriminative ability of ΔAHI for identifying normalization of insulin resistance (AUC = 0.62). **Conclusions:** Upper airway surgery was associated with significant improvements in insulin resistance and glycemic parameters in patients with OSA. The correlation between airway improvement and metabolic change supports a physiological link between upper airway obstruction and insulin sensitivity. These findings suggest that upper airway surgery may represent a clinically relevant adjunct within multimodal strategies for metabolic risk reduction, particularly in patients unable to tolerate CPAP therapy.

## 1. Introduction

Obstructive sleep apnea (OSA) is increasingly recognized as a major risk factor for the development of type 2 diabetes mellitus (T2DM) [1]. The prevalence of T2DM among individuals with OSA has been reported to range from 15% to 30%, markedly higher than the 8–10% observed in the general population [2,3]. Conversely, more than half of patients with T2DM may have undiagnosed OSA, with prevalence rates reaching up to 80% among those with coexisting obesity [4]. In addition to disease coexistence, OSA severity has been shown to correlate with poorer glycemic control, including higher glycated hemoglobin (HbA1c) levels [2]. These findings underscore the close and bidirectional relationship between OSA and metabolic dysfunction and support further investigation into the mechanistic links between sleep-disordered breathing and metabolic regulation.

The association between OSA and T2DM is mediated by multiple interrelated pathophysiological mechanisms, including intermittent hypoxemia, recurrent arousals, sleep fragmentation, and sympathetic nervous system activation [5,6]. Repeated episodes of upper airway obstruction led to intermittent hypoxemia, which induces oxidative stress and promotes insulin resistance through increased cortisol secretion, enhanced lipolysis, and elevated circulating free fatty acids [7,8]. Chronic hypoxemia has also been implicated in pancreatic β-cell dysfunction, further impairing insulin secretion [9,10]. In parallel, intermittent hypoxia and oxidative stress stimulate the release of pro-inflammatory cytokines, amplifying systemic inflammation and exacerbating insulin resistance [6]. Recurrent arousals and sleep fragmentation disrupt circadian regulation and reduce slow-wave sleep, both of which are essential for metabolic homeostasis [11]. Moreover, heightened sympathetic nervous system activity in OSA suppresses insulin secretion, alters hepatic glucose production, and promotes positive energy balance through increased appetite and reduced energy expenditure [12,13]. Collectively, these mechanisms support the concept that OSA is not merely a sleep-related breathing disorder but a systemic metabolic condition that actively contributes to insulin resistance and diabetes pathogenesis.

Beyond metabolic dysregulation, OSA imposes a profound broader cardiovascular burden. The condition is well-established as an independent risk factor for arterial hypertension, atrial fibrillation, coronary artery disease, and heart failure. Therefore, the metabolic improvements targeted by therapeutic interventions, such as reduced insulin resistance and improved fasting glycemia, occur within the context of a condition with severe cardiovascular implications. Surgical correction of OSA thus has potential benefits extending well beyond glycemic control, positioning upper airway surgery as part of a comprehensive approach to global cardiovascular and metabolic risk reduction.

Emerging evidence suggests that the relationship between OSA and metabolic dysfunction may differ according to sex. Compared with men, women with OSA often exhibit distinct clinical presentations, differences in upper airway anatomy and ventilatory control, and potential influences of sex hormones on metabolic regulation. Recent studies have further suggested that OSA-related cardiometabolic risk may manifest differently in women, highlighting the importance of considering sex-specific mechanisms when evaluating the metabolic consequences of sleep-disordered breathing.

Continuous positive airway pressure (CPAP) therapy remains the first-line treatment for OSA. Although CPAP effectively alleviates airway obstruction and improves nocturnal oxygenation, its long-term metabolic benefits remain uncertain [14]. Treatment adherence is highly variable, and suboptimal compliance substantially limits therapeutic effectiveness. While some studies have demonstrated short-term improvements in insulin sensitivity with CPAP therapy, long-term benefits in fasting glucose, HbA1c, or diabetes prevention have not been consistently observed [15,16,17,18,19,20]. To date, no clear consensus supports CPAP as an effective strategy for diabetes prevention in patients with OSA.

For patients who are unable or unwilling to tolerate CPAP therapy, upper airway surgery represents an alternative therapeutic approach targeting the anatomical basis of airway obstruction [21]. Unlike CPAP, which requires nightly adherence, surgical intervention provides a durable structural modification that may lead to sustained reductions in hypoxic burden and sleep fragmentation [22]. Recent large-scale observational studies suggest that surgically treated patients with OSA may exhibit a lower incidence of diabetes compared with those managed with CPAP alone [23]. These observations raise the possibility that upper airway surgery may confer metabolic benefits in addition to respiratory improvement, particularly in carefully selected patient populations.

Accordingly, this study was designed as a translational investigation to determine whether improvement in airway obstruction following upper airway surgery is associated with corresponding changes in insulin resistance. Specifically, we evaluated pre- and postoperative alterations in metabolic parameters—including fasting plasma glucose, insulin levels, HbA1c, and homeostatic model assessment for insulin resistance (HOMA-IR)—and examined whether the magnitude of surgical response correlated with metabolic improvement in patients with OSA.

## 2. Materials and Methods

### 2.1. Study Design and Participants

This retrospective study was conducted at a tertiary referral center. The study cohort included adult patients (≥20 years) with OSA who underwent upper airway surgery and had complete baseline and follow-up data between January 2024 and December 2025. All participants had polysomnography-confirmed OSA (AHI ≥ 5 events/hour) and were unable or unwilling to tolerate CPAP therapy.

Exclusion criteria were as follows: (1) age > 65 years; (2) morbid obesity (body mass index ≥ 37 kg/m^2^); (3) presence of advanced metabolic complications, including stroke, coronary heart disease, chronic kidney disease, neuropathy, retinopathy, or diabetic ketoacidosis; (4) use of medications known to affect sleep-disordered breathing (e.g., benzodiazepines, opioids, or muscle relaxants); (5) chronic systemic inflammatory diseases or endocrine disorders other than diabetes; and (6) use of medications known to significantly influence glucose metabolism.

### 2.2. Clinical and Metabolic Assessments

Baseline demographic characteristics, anthropometric measurements, and clinical data were extracted from electronic medical records. Body mass index (BMI) was calculated as weight in kilograms divided by height in meters squared (kg/m^2^). Polysomnographic variables included the AHI, mean oxygen saturation (SpO_2_), and nadir oxygen saturation.

Metabolic assessments were conducted preoperatively and six months postoperatively and included fasting plasma glucose, glycated hemoglobin (HbA1c), and fasting insulin levels. Insulin resistance was estimated using the homeostatic model assessment for insulin resistance (HOMA-IR). Incident diabetes at baseline was defined as fasting plasma glucose ≥ 126 mg/dL or HbA1c ≥ 6.5%, in accordance with established diagnostic criteria [24].

### 2.3. Surgical Intervention

All patients underwent individualized upper airway surgery following comprehensive preoperative airway assessments, including Friedman staging, lateral cephalometric analysis, and endoscopic evaluation [25,26,27]. Procedures were selected to correct site-specific obstruction at the nasal, palatal, and lingual levels [28,29,30,31] and were performed by a single experienced otolaryngologist. The surgical goal was anatomical stabilization of the upper airway to reduce obstructive respiratory events during sleep.

### 2.4. Outcome Measures

The primary outcome was change in insulin resistance, defined as the difference between postoperative and preoperative HOMA-IR (ΔHOMA-IR). Secondary outcomes comprised changes in glycemic indices, anthropometric measurements, and respiratory parameters, including fasting glucose, insulin levels, HbA1c, BMI, AHI, and oxygen saturation indices. BMI was evaluated to account for concurrent anthropometric variation potentially influencing metabolic outcomes.

### 2.5. Statistical Analysis

Continuous variables are presented as mean ± standard deviation, and categorical variables are expressed as counts and percentages. Paired *t*-tests were used to compare preoperative and postoperative clinical, respiratory, and metabolic parameters. Pearson correlation analysis was performed to assess the association between changes in AHI (ΔAHI) and changes in insulin resistance (ΔHOMA-IR).

Receiver operating characteristic (ROC) curve analysis was conducted to evaluate the ability of surgical response to predict postoperative normalization of insulin resistance, defined as HOMA-IR < 1.4 [32]. This cutoff value was selected based on commonly accepted thresholds for insulin resistance in non-diabetic populations [32]. The optimal discrimination threshold was determined using the Youden index. Given the limited sample size, multivariable regression analysis was not performed to avoid model overfitting. Accordingly, all observed associations should be interpreted as exploratory and hypothesis-generating rather than causal. Statistical analysis was performed using IBM SPSS Statistics (version 31.0; IBM Corp., Armonk, NY, USA). A two-tailed *p* value < 0.05 was considered statistically significant.

## 3. Results

### 3.1. Baseline Characteristics and Surgical Outcomes

A total of 49 patients with polysomnography-confirmed OSA were included in the analysis. The mean age was 41.6 ± 11.0 years, and 98% of participants were male. At baseline, the mean AHI was 46.6 ± 25.8 events/hour, with 91.8% of patients classified as having moderate-to-severe OSA. Baseline metabolic characteristics are presented in Table 1; the mean HOMA-IR was 2.8 ± 2.3, indicating a high prevalence of insulin resistance. Among the study cohort, 48 patients (98%) underwent multilevel airway surgery tailored to their specific anatomical pattern of upper airway obstruction identified during preoperative airway evaluation and DISE. Surgical procedures included combined nasal and palatal surgery in 7 patients (14%), nasal, palatal, and tongue surgery in 16 (33%), nasal, palatal, tongue, and epiglottic surgery in 12 (24%), and nasal, palatal, tongue, and genioplasty procedures in 13 (27%). Only one patient (2%) underwent isolated palatal surgery.

During six months following upper airway surgery, significant improvements were observed in respiratory parameters (Table 2). The AHI decreased from 46.6 ± 25.8 to 20.7 ± 14.1 events/hour (*p* < 0.001). Mean oxygen saturation increased from 92.2 ± 4.8% to 93.7 ± 1.9% (*p* = 0.015), and nadir oxygen saturation improved from 73.9 ± 13.0% to 80.1 ± 9.6% (*p* < 0.001), indicating effective reduction in upper airway obstruction during sleep.

### 3.2. Changes in Metabolic Parameters After Upper Airway Surgery

In parallel with respiratory improvement, significant postoperative changes were observed in metabolic parameters (Table 2). Fasting plasma glucose decreased from 105.5 ± 30.7 mg/dL to 93.8 ± 10.0 mg/dL (*p* = 0.014). Fasting insulin levels declined from 10.2 ± 6.5 to 7.2 ± 4.0 μU/mL (*p* = 0.003), and HOMA-IR decreased from 2.77 ± 2.29 to 1.68 ± 1.01 (*p* = 0.003). Although the reduction in HbA1c did not reach statistical significance, a downward trend was observed. Overall, these findings suggest that upper airway surgery was associated with improved insulin sensitivity.

### 3.3. Association Between Airway Improvement and Insulin Resistance

**Figure 1:**
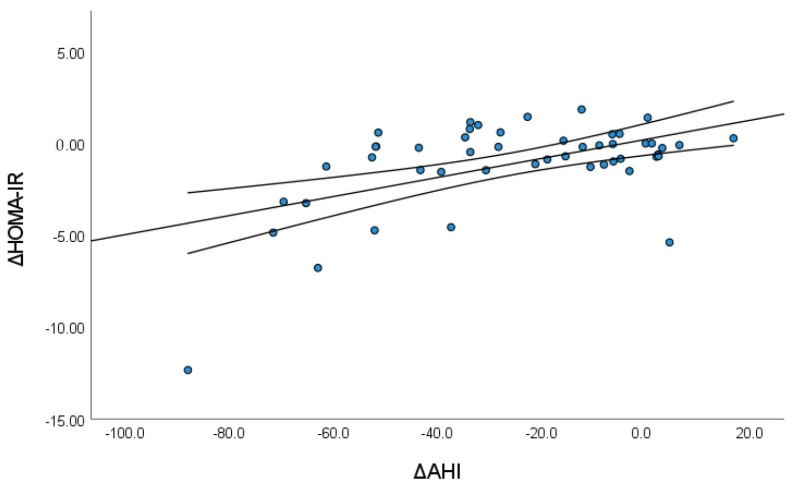
Correlation between ΔAHI and ΔHOMA-IR: Association between reduction in airway obstruction and improvement in insulin resistance. Scatter plot demonstrating a positive correlation between change in apnea–hypopnea index (ΔAHI) and change in HOMA-IR (ΔHOMA-IR) after upper airway surgery (r = 0.527, *p* < 0.001). The central solid line indicates linear regression, with the flanking lines representing the 95% confidence interval.

Correlation analysis demonstrated a moderate positive association between reduction in AHI (ΔAHI) and change in insulin resistance (ΔHOMA-IR) (r = 0.527, *p* < 0.001) (Figure 1). This finding suggests that greater reductions in airway obstruction were associated with larger decreases in insulin resistance following upper airway surgery, supporting a quantitative relationship between airway improvement and metabolic change.

### 3.4. Incident Diabetes Subgroup Analysis

**Figure 2:**
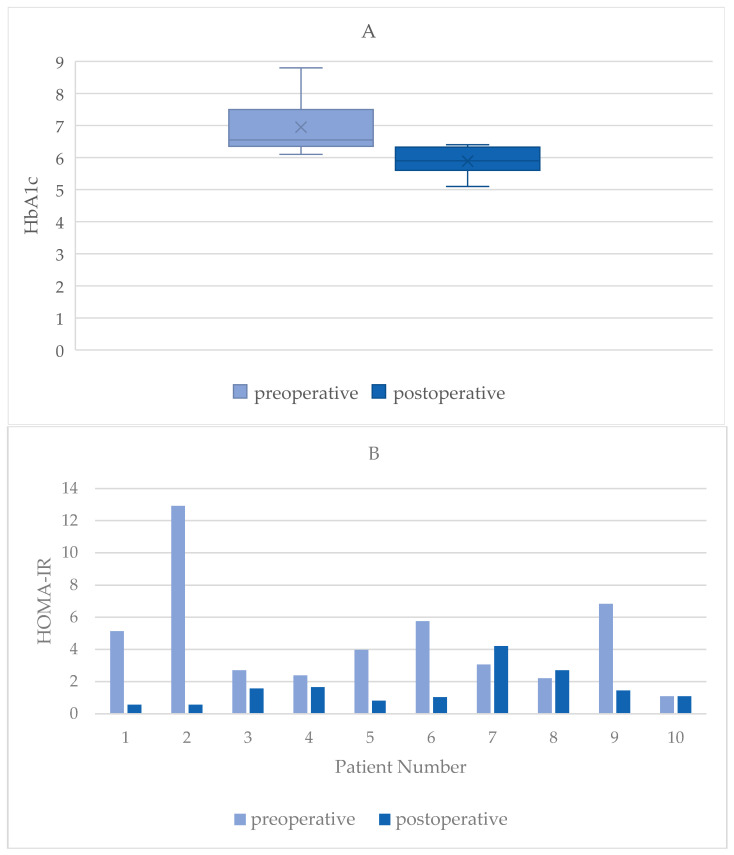
Pre- and postoperative glycemic indices in patients with diabetes at baseline. HbA1c normalized in all patients, whereas insulin resistance did not normalize universally. (**A**) Box-and-whisker plot shows median, interquartile range, and range. (**B**) Clustered bar chart shows individual patient values.

Among the 10 patients who met diagnostic criteria for diabetes at baseline, mean HbA1c decreased from 6.95 ± 0.93% preoperatively to 5.89 ± 0.44% postoperatively, and all patients achieved HbA1c values below the diagnostic threshold for diabetes at follow-up (Figure 2A). Notably, these individuals were identified incidentally through routine preoperative biochemical screening and had no history of antidiabetic medication use before or after surgery. However, normalization of insulin resistance (defined as HOMA-IR < 1.4) was not observed in all individuals, particularly among those with obesity (Figure 2B). The observation that HbA1c normalized in all diabetic patients whereas HOMA-IR remained elevated in some individuals suggests that HbA1c and HOMA-IR reflect different aspects of metabolic regulation. HbA1c reflects average glycemic control over the preceding 2–3 months, whereas HOMA-IR primarily reflects underlying insulin resistance and compensatory insulin secretion. Consequently, improvement in glycemic control may precede complete restoration of insulin sensitivity, particularly among patients with persistent obesity and ongoing adipose tissue-related metabolic dysfunction. These findings suggest that although upper airway surgery may be associated with improvement in early dysglycemia, it may not be sufficient as a standalone intervention to achieve complete metabolic normalization in higher-risk patients.

### 3.5. Predictive Value of Surgical Response for Insulin Resistance Normalization

**Figure 3:**
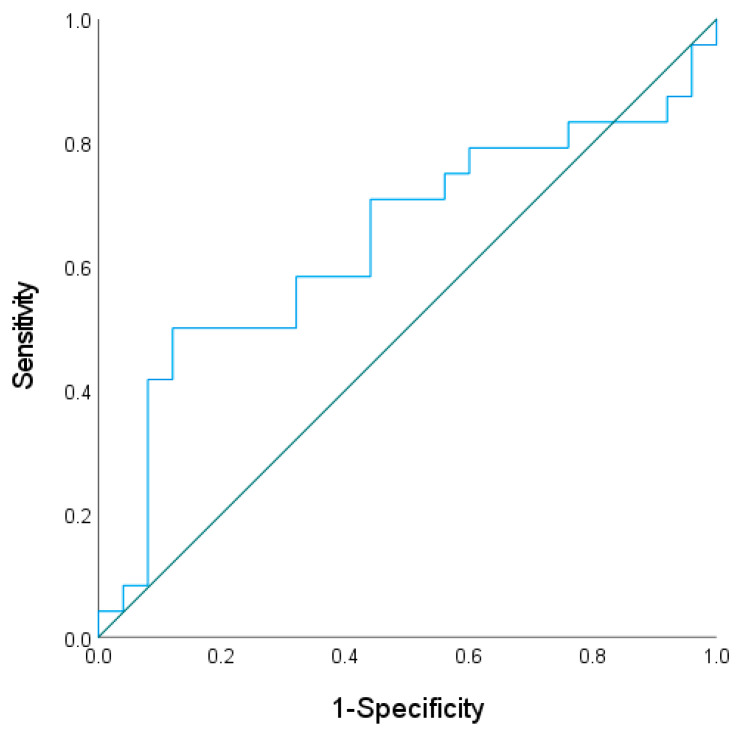
ROC analysis of ΔAHI and HOMA-IR normalization: Optimal ΔAHI threshold. The ROC curve for predicting postoperative HOMA-IR normalization is based on AHI reduction (ΔAHI). The optimal ΔAHI threshold was 37.1 events/h, with an area under the curve (AUC) of 0.62.

Receiver operating characteristic (ROC) analysis demonstrated modest discriminative ability of AHI reduction for predicting postoperative normalization of insulin resistance, with an area under the curve (AUC) of 0.62 (Figure 3). An AHI reduction of approximately 37 events/hour yielded the optimal cutoff for predicting HOMA-IR normalization. These findings suggest that greater reductions in AHI were associated with a higher likelihood of postoperative metabolic improvement.

## 4. Discussion

The present study demonstrates that upper airway surgery in OSA patients was associated with significant improvements in both respiratory parameters and insulin-related metabolic indices. In addition to marked reductions in AHI and improvements in oxygenation, postoperative decreases in fasting plasma glucose, fasting insulin levels, and HOMA-IR were observed. These findings suggest that surgical alleviation of upper airway obstruction may be accompanied by favorable changes in insulin sensitivity, supporting a physiological link between airway stabilization and metabolic regulation.

Previous studies evaluating metabolic outcomes after upper airway surgery have reported heterogeneous findings. Although improvements in inflammatory markers, lipid profiles, and cardiovascular risk factors have been consistently described, evidence regarding glycemic control and insulin resistance remains limited and inconsistent [23,33]. Several reviews have suggested that postoperative metabolic changes may be largely attributable to concomitant weight loss rather than airway modification alone [22,34]. In this context, the present study provides quantitative evidence demonstrating a moderate association between the magnitude of airway improvement and the degree of reduction in insulin resistance, supporting a physiological link between alleviation of obstructive respiratory events and metabolic response.

In this study, BMI decreased after surgery. Our correlation analysis revealed a positive association between ΔBMI and ΔHOMA-IR (r = 0.822, *p* < 0.001), underscoring that weight loss is a mediator of the observed metabolic improvement. While surgical alleviation of airway obstruction (ΔAHI) directly contributes to metabolic benefits by reducing intermittent hypoxemia and sleep fragmentation, the concurrent reduction in BMI cannot be overlooked. In our clinical practice, upper airway surgery is frequently complemented by postoperative lifestyle modifications, including dietitian-led education. This multidisciplinary approach likely creates a synergistic effect: surgical relief of airway obstruction improves sleep architecture and daytime energy levels, which in turn facilitates better adherence to dietary and lifestyle modifications. Together, the combined structural airway improvement and subsequent weight loss jointly drive the profound favorable changes in insulin sensitivity. Beyond these weight-mediated effects, from a mechanistic perspective, reductions in intermittent hypoxemia and sleep fragmentation also critically account for the metabolic improvements observed in this study. As previously noted, OSA is characterized by recurrent oxygen desaturation, oxidative stress, systemic inflammation, and sympathetic nervous system activation, all of which are known to impair insulin signaling and contribute to pancreatic β-cell dysfunction [6]. By restoring upper airway patency, upper airway surgery can reduce nocturnal hypoxic burden and improve sleep continuity, thereby potentially attenuating these adverse metabolic pathways [35,36].

Sex-related differences should also be considered when interpreting the present findings. Emerging evidence indicates that OSA pathophysiology, clinical manifestations, and associated cardiometabolic consequences may differ between men and women. Hormonal influences, body fat distribution, and differences in ventilatory control and upper airway physiology may modify the metabolic response to sleep-disordered breathing. Consequently, whether the association between airway improvement and insulin resistance observed in this predominantly male cohort can be generalized to women requires further investigation.

CPAP remains the first-line therapy for OSA and has been shown to produce modest short-term improvements in insulin sensitivity when adherence is adequate [37]. However, long-term metabolic benefits of CPAP remain inconsistent, largely owing to variable compliance and persistent sleep disruption [14]. In this context, upper airway surgery offers a durable anatomical intervention for patients who are unable or unwilling to tolerate CPAP therapy. [38] The present findings are consistent with prior longitudinal studies reporting a lower incidence of diabetes among surgically treated patients with OSA compared with those managed with CPAP, suggesting that surgical intervention may contribute to metabolic risk reduction in selected populations [23].

Particularly noteworthy was the observation that all patients meeting diagnostic criteria for diabetes at baseline achieved postoperative normalization of HbA1c despite the absence of glucose-lowering pharmacotherapy. Although based on a small subgroup, this finding raises the possibility that alleviation of OSA-related metabolic stress plus postoperative diabetes education may contribute to meaningful improvement in early diabetes and warrants further investigation in larger prospective studies.

Regarding patient selection, our ROC analysis identified an AHI reduction of approximately 37 events/hour as the optimal threshold for predicting HOMA-IR normalization. Clinically, this substantial required reduction implies that primarily patients with severe baseline OSA possess the capacity to achieve this magnitude of change. While the modest AUC indicates that ΔAHI alone cannot fully capture the complex, multifactorial nature of metabolic recovery, it provides valuable mechanistic insight. Future predictive models should incorporate composite variables to comprehensively identify ideal surgical candidates.

**Figure 4:**
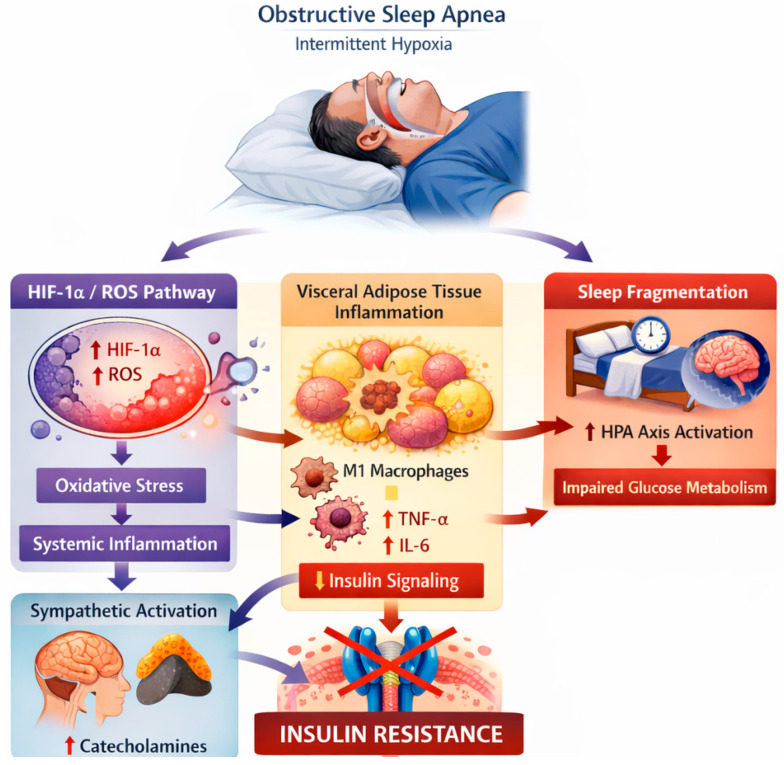
Molecular mechanisms linking obstructive sleep apnea to insulin resistance and diabetes, Intermittent hypoxia, inflammation, sympathetic activation, and sleep fragmentation in obstructive sleep apnea converge to impair insulin signaling, leading to insulin resistance, β-cell dysfunction, and hyperglycemia.

Beyond clinical observations, OSA contributes to insulin resistance and increased diabetes risk through several well-characterized molecular mechanisms. Intermittent hypoxia (IH) induces stabilization and activation of hypoxia-inducible factor-1α (HIF-1α), leading to upregulation of pro-oxidant enzymes and suppression of antioxidant gene expression [39,40]. Furthermore, IH induces a pro-inflammatory phenotype within visceral adipose tissue [41,42]. Sleep fragmentation independently disrupts glucose metabolism by increasing sympathetic activity and altering hypothalamic–pituitary–adrenal axis regulation [43,44]. Surgical interventions by upper airway procedures have been shown to decrease OSA severity and improve oxygen saturation, which mechanistically reduces intermittent hypoxia, sympathetic activation, and systemic inflammation—key drivers of insulin resistance in OSA [34,45,46] (Figure 4).

Several limitations should be acknowledged. First, the retrospective design and modest sample size limit causal inference and precluded multivariable adjustment. Second, lifestyle factors, including diet and physical activity, were not quantified. Third, the six-month follow-up period may be insufficient to assess the long-term durability of metabolic improvement. Fourth, although BMI decreased postoperatively, the relative contributions of weight change and airway stabilization to metabolic outcomes could not be fully disentangled. Finally, the single-center, single-surgeon design may limit generalizability, although it enhances procedural consistency and reduces inter-operator variability. In addition, the study population was predominantly male (98%), which may limit extrapolation of the findings to women, in whom OSA pathophysiology and metabolic consequences may differ.

## 5. Conclusions

Upper airway surgery in patients with OSA was associated with significant improvements in insulin resistance and glycemic parameters. The magnitude of metabolic improvement correlated with the degree of airway obstruction relief, supporting a physiological link between nocturnal breathing disturbance and insulin sensitivity. These findings suggest that upper airway surgery may represent a valuable adjunct within multimodal strategies for metabolic risk reduction in selected patients with OSA, particularly those unable to tolerate CPAP therapy. Future prospective studies are needed to determine whether surgical airway stabilization confers long-term protection against diabetes development.

## Figures and Tables

**Table 1:** Baseline characteristics of the study population (*n* = 49).

Variable	Value
Age, years	41.6 ± 11.0
Male sex, *n* (%)	48 (98.0)
Body mass index (kg/m^2^)	27.9 ± 5.4
Apnea–hypopnea index (events/h)	46.6 ± 25.8
Moderate–severe OSA, *n* (%)	45 (91.8)
Diabetes at baseline, *n* (%)	10 (20.4)
Family history of diabetes, *n* (%)	3 (6.1)
Hypertension, *n* (%)	10 (20.4)
Fasting plasma glucose (mg/dL)	105.5 ± 30.7
HbA1c (%)	5.91 ± 0.73
Fasting insulin (μU/mL)	10.2 ± 6.5
HOMA-IR	2.77 ± 2.29

Values are presented as mean ± standard deviation unless otherwise indicated. Abbreviations: OSA: obstructive sleep apnea; HbA1c: glycated hemoglobin; HOMA-IR: homeostatic model assessment for insulin resistance.

**Table 2:** Changes in respiratory and metabolic parameters before and after upper airway surgery.

Variable	Preoperative	Postoperative	*p* Value
Body mass index (kg/m^2^)	27.9 ± 5.4	26.4 ± 2.6	0.015
Apnea–hypopnea index (events/h)	46.6 ± 25.8	20.7 ± 14.1	<0.001
Fasting plasma glucose (mg/dL)	105.5 ± 30.7	93.8 ± 10.0	0.014
HbA1c (%)	5.91 ± 0.73	5.72 ± 0.37	0.077
Fasting insulin (μU/mL)	10.2 ± 6.5	7.2 ± 4.0	0.003
HOMA-IR	2.77 ± 2.29	1.68 ± 1.01	0.003
Mean SpO_2_ (%)	92.2 ± 4.8	93.7 ± 1.9	0.015
Nadir SpO_2_ (%)	73.9 ± 13.0	80.1 ± 9.6	<0.001

Values are presented as mean ± standard deviation. *p* values were calculated using paired *t*-tests. Abbreviations: HOMA-IR, homeostatic model assessment for insulin resistance.

## Data Availability

The data presented in this study are available on request from the corresponding author. The data are not publicly available due to strict privacy and ethical restrictions associated with patient clinical records and hospital policies.

## Abbreviations

The following abbreviations are used in this manuscript:

AHI	apnea–hypopnea index
AUC	area under the curve
BMI	body mass index
CPAP	continuous positive airway pressure
DM	diabetes mellitus
HbA1c	glycated hemoglobin
HIF-1α	hypoxia-inducible factor-1α
HOMA-IR	homeostatic model assessment for insulin resistance
HPA	hypothalamic–pituitary–adrenal
IH	intermittent hypoxia
IL-6	interleukin-6
OSA	obstructive sleep apnea
ROC	receiver operating characteristic
ROS	reactive oxygen species
SpO_2_	oxygen saturation
T2DM	type 2 diabetes mellitus
TNF-α	tumor necrosis factor-α
